# Si_3_C Monolayer as an Efficient Metal-Free Catalyst for Nitrate Electrochemical Reduction: A Computational Study

**DOI:** 10.3390/nano13212890

**Published:** 2023-10-31

**Authors:** Wanying Guo, Tiantian Zhao, Fengyu Li, Qinghai Cai, Jingxiang Zhao

**Affiliations:** 1 College of Chemistry and Chemical Engineering, and Key Laboratory of Photonic and Electronic Bandgap Materials, Ministry of Education, Harbin Normal University, Harbin 150025, China; guowanying@stu.hrbnu.edu.cn (W.G.); zhaotiantian@stu.hrbnu.edu.cn (T.Z.); caiqinghai@hrbnu.edu.cn (Q.C.); 2 School of Physical Science and Technology, Inner Mongolia University, Hohhot 010021, China

**Keywords:** nitrate electroreduction, metal-free catalysts, silicon carbide monolayer, synergistic effect, density functional theory

## Abstract

Nitrate electroreduction reaction to ammonia (NO_3_ER) holds great promise for both nitrogen pollution removal and valuable ammonia synthesis, which are still dependent on transition-metal-based catalysts at present. However, metal-free catalysts with multiple advantages for such processes have been rarely reported. Herein, by means of density functional theory (DFT) computations, in which the Perdew–Burke–Ernzerhof (PBE) functional is obtained by considering the possible van der Waals (vdW) interaction using the DFT+D3 method, we explored the potential of several two-dimensional (2D) silicon carbide monolayers as metal-free NO_3_ER catalysts. Our results revealed that the excellent synergistic effect between the three Si active sites within the Si_3_C monolayer enables the sufficient activation of NO_3_^−^ and promotes its further hydrogenation into NO_2_^*^, NO^*^, and NH_3_, making the Si_3_C monolayer exhibit high NO_3_ER activity with a low limiting potential of −0.43 V. In particular, such an electrochemical process is highly dependent on the pH value of the electrolytes, in which acidic conditions are more favorable for NO_3_ER. Moreover, ab initio molecular dynamics (AIMD) simulations demonstrated the high stability of the Si_3_C monolayer. In addition, the Si_3_C monolayer shows a low formation energy, excellent electronic properties, a superior suppression effect on competing reactions, and high stability, offering significant advantages for its experimental synthesis and practical applications in electrocatalysis. Thus, a Si_3_C monolayer can perform as a promising NO_3_ER catalyst, which would open a new avenue to further develop novel metal-free catalysts for NO_3_ER.

## 1. Introduction

The ever-increasing discharge of nitrate (NO_3_^−^)-containing agricultural and industrial sewage causes severe contamination of the soil and water resources, thus posing great threats to global food safety and human health [1,2]. At present, state-of-the-art nitrate treatment of wastewater typically involves the biological and physical strategies of denitrification, which have shown some intrinsic drawbacks [3,4,5]. For example, biological methods require denitrifying bacteria to convert NO_3_^−^ into nitrogen, which would be ineffective under extreme conditions, and the biomass materials would need to be post-treated [6]. As for the physical approaches, such as reverse osmosis and ion exchange, they are still focused on displacement rather than elimination, which requires further processing [7]. Chemical reduction is an interesting way to convert NO_3_^−^ into some desirable products, which can be driven using heat, light, and electrical energy [8]. Among them, the electrochemical reduction of NO_3_^−^ to ammonia reaction (NO_3_ER) has recently emerged as a most promising strategy for water denitrification due to the use of renewable electric energy, mild operation conditions under room temperature and pressure, and without secondary treatment [9,10,11,12]. More importantly, the NH_3_ production from this process can serve as a low energy-consuming alternative to the Haber−Bosch process [13,14,15]. Especially, compared with the nitrogen reduction reaction (NRR), NH_3_ synthesized from NO_3_^−^ is more preferable kinetically due to the higher water solubility of NO_3_^−^ and the lower dissociation energy of the N=O bond of NO_3_^−^ than that in a N_2_ molecule [16].

However, NO_3_^−^-to-NH_3_ conversion is a very complex process, as it involves the transfer of eight electrons and nine protons, during which NO, NO_2_, N_2_O_2_, and N_2_ could be produced as undesired byproducts [17,18,19]. In addition, the competitive hydrogen evolution reaction (HER) renders the NO_3_ER less selective. Thus, efficient NO_3_ER catalysts are urgently required. So far, transition-metal-based electrocatalysts, such as alloy [20,21,22], metal oxide [23,24], and singe atom catalysts [25,26,27], have been extensively investigated and achieved a Faradaic efficiency (FE) higher than 90% for NH_3_ synthesis from the NO_3_ER process. However, the scarcity, high price, and the instability of metal under harsh working conditions greatly hamper its large-scale application and could cause environmental pollution [28]. In this regard, metal-free electrocatalysts exhibit multiple advantages, including their earth abundance, low cost, environment friendliness, long durability, and transformable structures [29,30]. Thus, the development of *metal-free* catalysts for NO_3_^−^ electroreduction into a NH_3_ product is of great significance [31,32].

In recent years, various two-dimensional (2D) materials have been widely studied to develop efficient electrocatalysts for some electrochemical reactions due to their unique structure and electronic properties [33,34,35]. Among them, 2D silicon carbides (Si*_x_*C*_y_*) are of particular interest due to their rich stoichiometries and tunable electronic properties [36]. For example, the SiC, SiC_2_, and SiC_7_ monolayers exhibit semiconducting features with band gaps of 2.55 [37], 1.09 [38], and 1.13 eV [39], respectively, and the SiC_3_ monolayer is a topological insulator [40]. On the contrary, the Si_3_C and SiC_5_ monolayers are semi-metal materials [41,42]. These intriguing properties endow 2D Si*_x_*C*_y_* nanomaterials with wide applications in gas sensors [43], Li-ion batteries [44,45,46,47], and catalysis [48,49,50,51,52,53].

With these advantages of 2D Si*_x_*C*_y_* materials in mind, here, we investigated the feasibility of several Si*_x_*C*_y_* materials (including SiC, SiC_2_, SiC_3_, SiC_5_, SiC_7_, and Si_3_C monolayers) as catalysts for NO_3_ER into NH_3_ by means of density functional theory (DFT) computations. The results demonstrated that, among these Si*_x_*C*_y_* candidates, the Si_3_C monolayer can be identified as the best NO_3_ER catalyst due to its lowest limiting potential of −0.43 V, which stems from the synergic effect of its three adjacent Si active sites to boost the efficient activation of NO_3_^−^ and the subsequent hydrogenation step. Moreover, the positive charge on Si active sites in the Si_3_C monolayer greatly hinders H^+^ attacking, guaranteeing its high selectivity toward NO_3_ER by suppressing the competing HER. In addition, the high stability of the Si_3_C monolayer can be verified from a thermal and electrochemical perspective to meet its realistic applications in NO_3_ER.

## 2. Computational Methods and Models

All spin-polarized DFT computations were carried out by using a plane-wave basis set, as implemented in the Vienna Ab Initio Simulation Package (VASP 5.4.1) [54,55], in which the projector augmented wave (PAW) potential was adopted for the interactions between electrons and ions [56,57], and the Perdew–Burke–Ernzerhof (PBE) functional [58] within the generalized gradient approximation (GGA) was employed to determine the exchange–correlation interactions with a cutoff energy of 550 eV. The convergence criteria were set to 0.01 eV/Å and 10^−5^ eV, respectively, for the residual force and the energy on each atom during structure formation. The possible van der Waals (vdW) interaction was treated using the empirical correction in Grimme’s method (DFT+D3) [59].

A supercell consisting of 3 × 3 × 1 Si*_x_*C*_y_* unit cells was built with a vacuum space of 20 Å to minimize the interaction between two adjacent images. During the structural relaxation, a 3 × 3 × 1 *k*–point mesh was employed to sample the 2D Brillouin zone. Ab initio molecular dynamics (AIMD) simulations based on the NVT ensemble [60] were performed to evaluate the thermodynamic stability. To explore the catalytic activity of these Si*_x_*C*_y_* monolayers for NO_3_ER, the computational electrode model (CHE) method [61,62] was employed to compute the free energy diagrams and the corresponding limiting potentials (U_L_). Specially, the free energy change (∆G) of each elementary step can be determined by ∆G=∆E+∆ZPE−T∆S+eU, where ∆E is the reaction energy of the reactant and product species adsorbed on the catalyst, directly obtained from DFT computations, and ∆ZPE and ∆S (see Table S1 in Supporting Information) represent the differences in the zero-point energy and entropy of the adsorbed species and the gas phase molecules at 298.15 K, respectively, which can be calculated from the vibrational frequencies. Specially, the entropies of the free molecules (H_2_, H_2_O, and NH_3_) were taken from the NIST database [63], whereas the energy contribution from the configurational entropy in the adsorbed intermediate was neglected. U represents the applied potential, which can be determined as U = −∆G/e. Thus, U_L_ corresponds to the applied limiting potential, which can be obtained using the maximum free energy change (∆*G_max_*) to overcome in the NO_3_ER process: UL = −∆*Gmax*/*e*, well consistent with previous studies [64,65]. To avoid directly computing the energy of NO_3_^−^, the gaseous HNO_3_ was chosen as a reference. The Gibbs adsorption free energy of NO_3_^−^ ($∆G_{NO_{3}^{*}}$) can be computed by$\;∆G_{NO_{3}^{*}}=G_{NO_{3}^{*}}-G_{*}-G_{HNO_{3}\left(g\right)}+\frac{1}{2}G_{H_{2}\left(g\right)}+{∆G}_{correct}$, where $G_{NO_{3}^{*}}$, $G_{*}$, $G_{HNO_{3}\left(g\right)}$, and $G_{H_{2}\left(g\right)}$ represent the Gibbs free energies of the adsorbed NO_3_^−^, Si*_x_*C*_y_* catalyst, gas-phase HNO_3_, and H_2_ molecules, respectively, where ${∆G}_{correct}$ is the correction for the NO_3_^−^ adsorption energy, which was set to 0.392 eV based on previous theoretical studies [66,67,68,69,70,71,72,73].

## 3. Results and Discussion

### 3.1. Structures and Properties of 2D Si_x_C_y_ Nanomaterials

**Figure 1:**
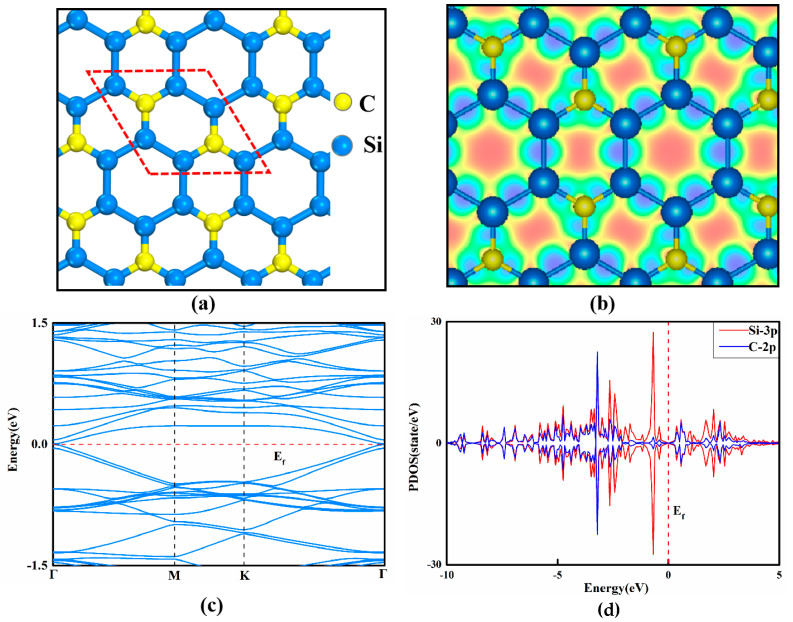
(**a**) Optimized structure of Si_3_C; (**b**) charge density distribution of Si_3_C monolayer (the purple and green areas represent positive and negative charges, respectively); (**c**) band structure of Si_3_C; (**d**) projected density of states (PDOSs) of Si_3_C monolayer. The Fermi level was set to zero for red dotted lines.

The optimized structures of these considered 2D Si*_x_*C*_y_* nanomaterials are presented in Figure 1a and Figure S1, while some key parameters are summarized in Table S2. Clearly, all Si*_x_*C*_y_* monolayers have a graphene-like planar structure, which is unlike the puckered structure of silicene. Moreover, the optimized lattice parameters of their unit cells are in the range of 3.09 (SiC) to 7.02 Å (Si_3_C), and the lengths of the Si–C bonds range from 1.69 Å (SiC_7_) to 1.81 Å (SiC_3_ and Si_3_C). In particular, within the frameworks of the SiC_2_, SiC_3_, SiC_5_, and SiC_7_ monolayers, some C–C bonds can be formed with shortest lengths of about 1.44 Å. However, in the Si_3_C monolayer, the Si–Si bonds can be formed with lengths of 2.45 Å. Notably, the above results on the optimized configurations of these Si*_x_*C*_y_* nanomaterials are in good agreement with previous studies [41,45,74] as shown in Table S3, thus ensuring the accuracy of the employed models and methods. Remarkably, the planar configurations of these Si*_x_*C*_y_* monolayers could be related to the ionic-binding features of the Si-C bonds, as shown by the computed charge density distribution in Figure 1b and Figure S1. As expected, due to the larger electronegativity of the C atom than that of the Si atom, there is a significant amount of charge transfer (0.74~2.51|e^–^|) from the Si atom to the C atom. As a result, the Si atom carries the positive charge, making it exhibit similar electronic properties to the transition metal and thus holding great potential for efficiently activating NO_3_^–^ in NO_3_ER.

To estimate the experimental feasibility of these 2D Si*_x_*C*_y_* materials, we computed their formation energies (*E*_f_) under C-rich and Si-rich conditions according to the following definition: $E_{f}=(E_{total}-n_{C}\times \mu_{C}-n_{Si}\times \mu_{Si})/(n_{C}+n_{Si})$, where $E_{total}$ is the total electronic energy of a Si*_x_*C*_y_* monolayer, and $n_{C}$ and $n_{Si}$ are the number of C and Si atoms in the supercell of the Si*_x_*C*_y_* monolayer according to previous theoretical studies [40]. Moreover, μ_C_ and μ_Si_ represent the chemical potentials of the C and Si atoms, which greatly depend on the environment conditions. Under C-rich conditions, μ_C_ was computed from the graphene, and then the chemical potential of the Si atoms was determined by μ_Si_ = μ_SiC_ − μ_C_, where μ_SiC_ denotes the chemical potential of a SiC unit cell in bulk SiC crystal. On the contrary, the cubic silicon crystal was adopted as the source of Si atoms, and μ_C_ can be obtained as follows: μ_C_ = μ_SiC_ − μ_Si_. Based on the above definitions, the computed *E*_f_ values of these 2D Si*_x_*C*_y_* systems were summarized in Table S2. Our DFT results showed that the *E*_f_ of the SiC monolayer is 0.67 eV, which is independent of the growth conditions due to the same ratio between the Si and C atoms. In contrast, the SiC_2_, SiC_3_, SiC_5_, and SiC_7_ candidates exhibit lower formation energies (0.52~0.66 eV) in C-rich environments than those in Si-rich ones (0.86~1.04 eV), suggesting that such conditions can boost their formation. On the contrary, the Si_3_C monolayer prefers to grow in Si-rich conditions due to its smaller *E*_f_ value of 0.89 eV. These positive formation energies suggest the synthesis of these Si*_x_*C*_y_* materials still remains challenging in the experiments. Fortunately, rapid progress in the growth of 2D materials on metal-based surfaces by using chemical vapor deposition has been made in recent years [75,76]. For example, Polley et al. reported the synthesis of monolayer honeycomb SiC on ultrathin metal carbide films [77], and Gao et al. synthesized a Si_9_C_15_ monolayer on Ru substrates [78]. In addition, the reaction between graphene and a Si source is another promising synthetic strategy for 2D Si*_x_*C*_y_* materials, such as a quasi-2D SiC_2_ monolayer [79].

The electrical conductivity of a given catalyst has been revealed as an important indicator to evaluate its electrocatalytic activity. In general, excellent electrical conductivity normally facilitates rapid charge transfer for an efficient electrochemical process. Therefore, the band structures of these 2D Si*_x_*C*_y_*candidates were computed to estimate their electrical conductivity. We found that the SiC, SiC_2_, or SiC_7_ monolayers are semiconductors with large band gaps of 2.55, 0.61, and 0.76 eV (Figure S1), respectively, which would be unfavorable for charge transfer for electrocatalytic reactions. On the contrary, analysis of the band structure shows that SiC_3_ (Figure S1) and Si_3_C monolayers (Figure 1c) are semi-metallic with the conduction band minimum (CBM) and VBM contacting each other at the point of Γ(k = 0) to form a Dirac cone, implying their good electrical conductivity to boost their applications in electrocatalysis, which mainly originates from the contributions of Si-3p orbitals, as shown by the computed projected density of states (PDOSs, Figure 1d).

### 3.2. Adsorption and Activation of NO_3_^−^ on Si_x_C_y_ Monolayers

**Figure 2:**
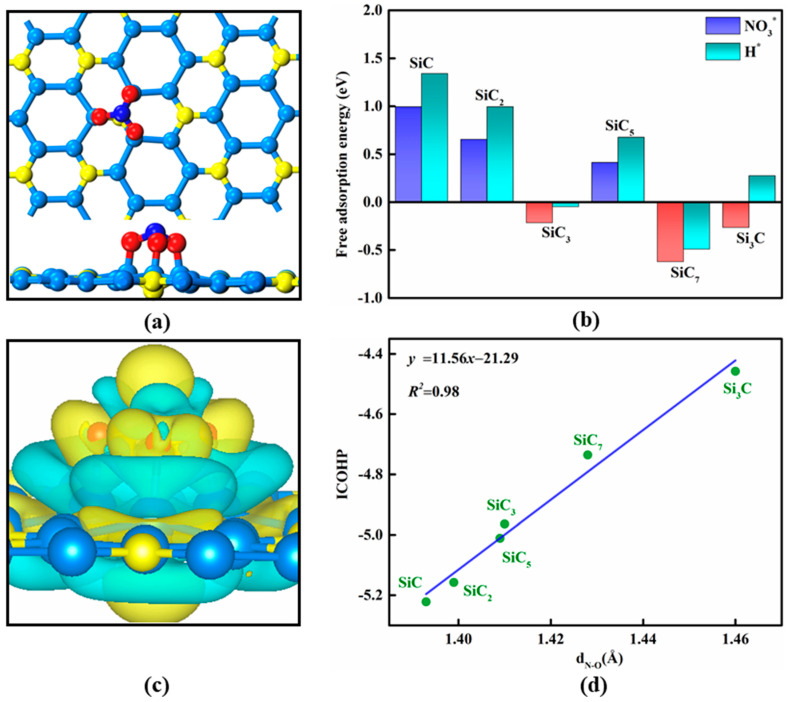
(**a**) Optimized NO_3_^−^ adsorption structure on Si_3_C monolayer; (**b**) the computed free adsorption energies of NO_3_^−^ and H^+^ on Si*_x_*C*_y_* monolayers; (**c**) charge density differences (∆ρ) of NO_3_^−^ adsorption on Si_3_C monolayer with an isosurface of 0.003 e Å^−3^ (cyan and yellow bubbles denote charge depletion and accumulation, where ∆ρ = ρ_NO3*_ − ρ_*_ − ρ_NO3_, in which ρ represents the charge density of a given material); (**d**) the correlation between ICOHP and the distance of N-O bond in the adsorbed NO_3_^*^ (d_N-O_).

During the process of NO_3_^−^ electroreduction, it is well established that the first step is the adsorption of the nitrate species, which often affects and even determines the whole catalytic reaction pathway. To this end, we next examined the adsorption behavior of the NO_3_^−^ species on these 2D Si*_x_*C*_y_* candidates. To obtain the most stable adsorption structure, three possible initial configurations were taken into account, including 1–O, 2–O, and 3–O patterns, in which the NO_3_^−^ species is adsorbed on the Si or C active sites via its one, two, or three O atoms. After full structural relaxation, the obtained most stable NO_3_^−^ adsorption configurations for these Si*_x_*C*_y_* candidates are shown in Figure 2a and Figure S2, and the computed Gibbs adsorption free energies are summarized in Figure 2b.

The results showed that NO_3_^−^ is preferable to be adsorbed on SiC, SiC_2_, SiC_3_, SiC_5_, and SiC_7_ systems via the 1–O pattern, in which one Si–O bond is formed with a length ranging from 1.74 to 1.79 Å. On a Si_3_C monolayer, however, we found that the three O atoms of NO_3_^−^ species can be adsorbed on three Si sites of this catalyst, forming three Si–O bonds with lengths of 1.74 Å (Figure 2a). Notably, to the best of our knowledge, there is no prior study on such a NO_3_^−^ adsorption configuration via the 3–O pattern, which mainly stems from the unique geometric ensemble effect in the Si_3_C monolayer: its three adjacent Si active sites can promote synergistically the sufficient activation of the NO_3_^−^ species.

Based on these aforementioned adsorption configurations, we then evaluated the binding strength of NO_3_^−^ on these 2D Si*_x_*C*_y_* catalysts by computing their corresponding $∆G_{NO_{3}^{*}}$ values. Unfortunately, NO_3_^−^ physisorption can be observed on the SiC, SiC_2_, and SiC_5_ monolayers due to their computed positive $∆G_{NO_{3}^{*}}$ values (0.99 eV, 0.65 eV, and 0.41 eV, respectively, Figure 2b), suggesting that NO_3_^−^ cannot be effectively captured by these three Si*_x_*C*_y_* materials, let alone effectively activated. We thus excluded them as promising NO_3_ER catalysts from further studies in this work. Conversely, the spontaneous chemisorption of NO_3_^−^ can be achieved on the SiC_3_, SiC_7_, and Si_3_C monolayers with $∆G_{NO_{3}^{*}}$ values of −0.21, −0.62, and −0.26 eV, respectively. Moreover, to suppress the unwanted hydrogen evolution reaction (HER) for achieving a high selectivity, the $∆G_{NO_{3}^{*}}$ value should be more negative than that of the H^+^ species ($∆G_{H^{*}}$), since the H^+^ in the electrolytes will block the active sites when H^+^ binds too strongly with a given catalyst. Notably, a comparison between $∆G_{H^{*}}$ and $∆G_{NO_{3}^{*}}\;$has been extensively employed to estimate the selectivity of a given catalyst for NO_3_ER [65,66,67,68,69,70,71,72,73]. To this end, the values of $∆G_{NO_{3}^{*}}$ and $∆G_{H^{*}}$ on the SiC_3_, SiC_7_, and Si_3_C surfaces are presented in Figure 2b for comparison. Clearly, the $∆G_{NO_{3}^{*}}$ values are more negative than the corresponding $∆G_{H^{*}}$ in the three candidates, especially for the Si_3_C monolayer (−0.26 eV for $∆G_{NO_{3}^{*}}$ vs. 0.27 eV for $∆G_{H^{*}}$), indicating the good suppressing effect on the undesirable HER and thus ensuring a high selectivity toward the NO_3_ER process. Understandably, the positive charges on the Si active sites can greatly hamper H^+^ approaching and H^*^ formation due to the electrostatic repulsion between the positively charged Si sites and H^+^.

To gain a deep insight into the NO_3_^−^ chemisorption, we took the Si_3_C monolayer as an example to compute the corresponding charge density differences (Figure 2c). The results show that a considerable amount of negative charge is accumulating between the adsorbed O atoms of the NO_3_^*^ species and Si active sites. According to Bader charge analysis, about 2.30 electrons are transferred from the p-orbitals of the three Si active sites to the empty π^*^-orbital of the NO_3_^−^ species, resulting in the sufficient activation of the adsorbed NO_3_^−^ species via the p–π^*^ interaction, which normally helps trigger the subsequent hydrogenation reaction. In addition, to explain the remarkable difference in the adsorption strength of NO_3_^−^ in these 2D Si*_x_*C*_y_* materials, integrated crystal orbital Hamilton population (ICOHP) analyses of the adsorbed NO_3_^*^ species were computed. Here, a more negative ICOHP value implies a less activated NO_3_^*^ species. As displayed in Figure S3, for the Si_3_C monolayer, there is a strong orbital interaction with NO_3_^*^ due to the fully occupied bonding orbitals and nearly unoccupied antibonding orbitals, inducing weaker N=O bonding and more sufficient NO_3_^*^ activation, which can be also confirmed by its less negative ICOHP. Interestingly, a good linear correlation can be observed between the ICOHP and the N–O bond lengths of the adsorbed NO_3_^*^ species (Figure 2d), well accounting for the NO_3_^−^ adsorption trend in these Si*_x_*C*_y_* catalysts, because their different Si active sites determine different bonding/anti-bonding orbital populations.

### 3.3. Catalytic Performance of SiC_3_, SiC_7_, and Si_3_C Monolayers for NO_3_ER

**Figure 3:**
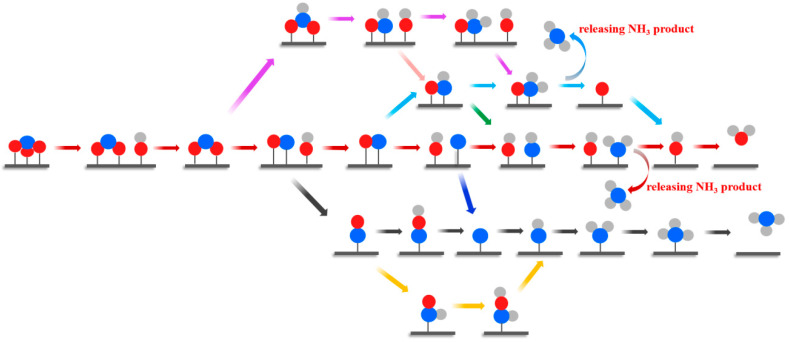
The considered possible reaction pathways for NH_3_ synthesis from NO_3_ER on Si_3_C monolayer.

Since sufficient NO_3_^–^ activation has been confirmed on the SiC_3_, SiC_7_, and Si_3_C monolayers, we further evaluated their NO_3_ER catalytic performance by computing the free energy changes (ΔG) of all possible elemental steps, which was presented in Figure 3 according to a summary of previous studies on NO_3_ER [65,66,67,68,69,70,71,72,73]. Specially, by computing the ΔG values, we can identify the reaction pathway with the lowest positive free energy change between any two elementary steps, namely, the most favorable reaction pathway.

**Figure 4:**
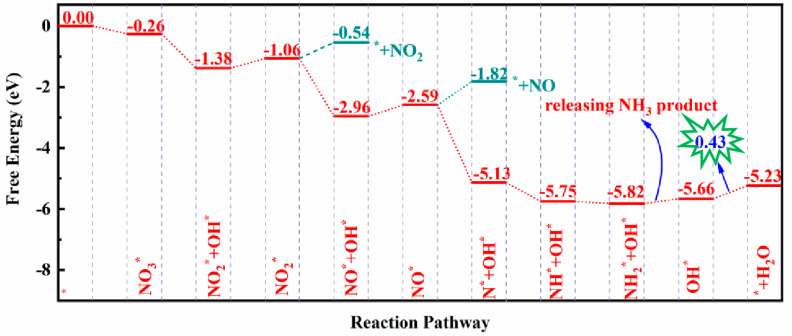
The computed free energy profile for NH_3_ synthesis from NO_3_ER on Si_3_C monolayer.

For simplicity, we again chose the Si_3_C monolayer as an example to elaborate the whole NO_3_ER process. The obtained most favorable reaction pathway and the involved configurations are shown in Figure 4 and Figure S4, while the computed ΔG values of other possible elementary steps are summarized in Table S4. It can be seen from Figure 4 that NO_3_^−^ can be stably adsorbed on three Si sites by forming three Si–O bonds with a ΔG value of −0.26 eV. Interestingly, the approach of the first (H^+^ + e^−^) pair leads to the dissociation of one N–O bond of NO_3_^*^ to generate (NO_2_^*^ + OH^*^) species, which is highly downhill by 1.12 eV in the free energy profile. Kinetically, however, this step of NO_3_^*^ → NO_2_^*^ + OH^*^ requires crossing an energy barrier of 0.95 eV (Figure S5). Subsequently, a H_2_O molecule can be released via OH^*^ hydrogenation, which is slightly endothermic by 0.32 eV. After the formation of H_2_O, the remaining NO_2_^*^, bound to two Si sites with Si–O lengths of 1.79 Å, can react with another (H^+^ + e^−^). Due to the synergistic effect of the Si active sites, the hydrogenation of the NO_2_^*^ intermediate also induces the cleavage of one N–O bond spontaneously, forming a (NO^*^ + OH^*^) group. Remarkably, this step of (NO_2_^*^ + H^+^ + e^−^ → NO^*^ + OH^*^) is highly exothermic by 1.90 eV. Next, an endothermal process for NO^*^ formation and H_2_O desorption is observed with the free energy increased by 0.37 eV. Again, when the H proton attacks the remaining NO^*^ intermediate, its N–O bond is split into (N^*^ + OH^*^) with a considerable negative ΔG of −2.54 eV, in which the N atom is adsorbed on the Si–Si bridge site with a length of 1.65 Å. In the subsequent steps, the H proton consecutively attacks the N^*^ intermediates to generate (NH^*^ + OH^*^) and (NH_2_^*^ + OH^*^) with ΔG values of −0.62 and −0.07 eV, respectively. Moreover, the NH_2_^*^ species continues to be hydrogenated to achieve a NH_3_ product, and this step is slightly exothermic by 0.16 eV. Eventually, the residual OH^*^ species is reduced to H_2_O, and the corresponding energy rises by 0.43 eV.

Overall, the most energetically favorable conversion process from NO_3_^−^ into NH_3_ product on the surface of the Si_3_C monolayer can be summarized as follows: NO_3_^−^ → NO_3_^*^ → NO_2_^*^ + OH^*^ → NO_2_^*^ → NO^*^ + OH^*^ → NO^*^→ N^*^ + OH^*^ → NH^*^ + OH^*^ → NH_2_^*^ + OH^*^ → OH^*^ →H_2_O (Figure 4), in which the reduction of OH^*^ into H_2_O can be identified as the potential-determining step (PDS) due to its maximum ΔG value of 0.43 eV. Thus, the U_L_ of NO_3_ER on the Si_3_C monolayer is computed to be −0.43 V, which is comparable (even less negative) to some transition metal-based catalysts (–0.34 to –0.53 V) [63,64,65,66,67,68,69], suggesting the high NO_3_ER catalytic activity of the Si_3_C monolayer. In addition to the Si_3_C monolayer, we also estimated the activity of the SiC_3_ and SiC_7_ monolayers for NO_3_ER. We found that the NO_3_ER process on the two materials is also hampered by OH^*^ desorption (Figure S6), which requires a high energy input of 0.79 and 1.23 eV, respectively, corresponding to more negative U_L_ values of −0.79 and −1.23 V than that of Si_3_C system (−0.43 V). Of note, to examine the accuracy of the employed PBE function in predicting the catalytic activity, we re-computed the free energy profile of NO_3_ER by using the revised PBE functional (rPBE), in which Si_3_C was chosen as an example. Fortunately, we found that the difference in the limiting potential between the two methods is nearly negligible (0.01 V, Figure S7), thus validating the high activity of the Si_3_C monolayer toward NO_3_ER.

Another important issue is the selectivity of the Si_3_C monolayer toward NO_3_ER. We thus examined the reaction pathways to form some N-containing byproducts, including NO_2_, NO, and N_2_. It can be seen from Figure 4 and Figure S8 that the free energy barriers for the release of NO_2_, NO, and N_2_ are 0.52, 0.77, and 0.54 eV, respectively, which are much larger than that of NH_3_ desorption (0.16 eV). Thus, it is rather difficult to generate these N-based byproducts on the Si_3_C monolayer, indicative of its high selectivity toward NH_3_ production from NO_3_ER. In addition, the HER can be well suppressed due to the weaker H* adsorption on the Si active sites than NO_3_^−^ (−0.26 eV for $∆G_{NO_{3}^{*}}$ vs. 0.27 eV for $∆G_{H^{*}}$) as in the above discussion (Figure 2b).

### 3.4. pH-Dependent NO_3_ER Activity

**Figure 5:**
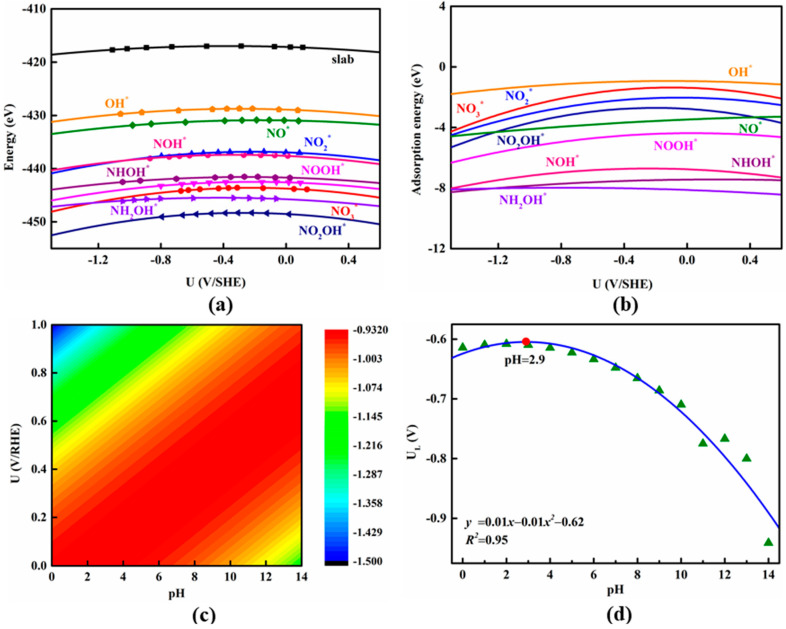
(**a**) Electronic energies of Si_3_C monolayer with and without the adsorbed NO_3_ER intermediates as a function of the applied electrode potential; (**b**) adsorption energies of various NO_3_ER intermediates as a function of the applied electrode potential; (**c**) pH-dependent and potential-dependent contour plot of adsorption energies of OH^*^ on Si_3_C monolayer; (**d**) the limiting potentials (U_L_) of NO_3_ER as a function of pH value.

It should be noted that NO_3_ER generally proceeds in aqueous conditions, which could induce pH-dependent activity in a given electrocatalyst. To examine this concern, the constant potential method (CPM; more computational details can be seen in ESI) developed by Duan et al. [80,81,82,83,84] was employed to explore the pH effects on the NO_3_ER catalytic activity on the Si_3_C monolayer. The variations of the total electronic energies of the Si_3_C monolayer with and without the adsorbed NO_3_ER intermediates with the applied electrode potential (standard hydrogen electrode, SHE) are presented in Figure 5a, while the corresponding fitted data are summarized in Table S5, from which a good quadratic relation for all these energy potential points can be observed. Moreover, the adsorption energies (*E*_ads_; more computational details are provided in Supporting Information) of these reaction intermediates at various applied potentials were computed as shown in Figure 5b. As the OH^*^ desorption was identified as the potential-determining step in NO_3_ER, its binding strength with the Si_3_C monolayer at different pHs and applied potentials is crucial to determine its pH- and potential-dependent activity. The computed *E*_ads_ values of the OH^*^ species as function pHs and applied potentials are plotted in Figure 5c. The results indicated that alkaline conditions undoubtedly hinder the PDS process due to the stronger OH^*^ adsorption strength on the Si_3_C monolayer, suggesting the obvious pH-dependent NO_3_ER activity of the Si_3_C monolayer. As a result, the Si_3_C monolayer always exhibits a higher catalytic activity in acidic solutions (reflected by a lower limiting potential) than that in alkaline ones (Figure 5d). In particular, when the pH is about 2.90, the OH^*^ adsorption is optimal, thus achieving the highest NO_3_ER activity with the lowest limiting potential of −0.61 V vs. the reverse hydrogen electrode (RHE) and the involved free energy profile presented in Figure S9. Noteworthily, highly efficient NO_3_ER can be usually achieved in neutral and alkaline media so far. Yet, there are only a few reports on efficient NO_3_ER in acidic NO_3_^−^-containing wastewater [85], which can meet the requirements of industrial processes such as mining, metallurgy, metal processing, and petrochemical and fiber engineering. Especially as compared with neutral/alkaline conditions, NO_3_ER in acidic conditions is more beneficial for directly generating fertilizers (such as ammonium sulphate, ammonium chloride, etc.) from a chemical perspective and meanwhile avoiding the volatilization of NH_3_ gas from aqueous ammonia in neutral/alkaline products [86,87].

### 3.5. Stability of Si_3_C Monolayer

Stability is a prerequisite for the practical applications of a catalyst. As a result, we evaluated the durability of the Si_3_C monolayer by performing ab initio molecular dynamics (AIMD) simulations. The results demonstrated that the geometric structure of the Si_3_C monolayer can be well preserved at 500 K after 10 ps (Figure S10a), suggesting its excellent thermal stability. In addition, we also examined the Poisson probability of the bare surfaces of the Si_3_C monolayer using the O^*^/OH^*^ species stemming from the aqueous solution under working conditions. Thus, we constructed the surface Pourbaix profile [88,89,90] of the Si_3_C monolayer to examine its surface configurations under different equilibrium potentials and pH values. As shown in Figure S10b, when the electrode potential is 0 V vs. SHE, the basal plane of the Si_3_C monolayer is only covered by OH^*^ species in an alkaline condition. Moreover, the minimum potential required to remove the surface OH^*^ group on the Si_3_C monolayer at pH = 14 (U_R_) is −0.39 V, which is less negative than the U_L_ of NO_3_ER (−0.43 V), indicating the superior electrochemical stability of the Si_3_C monolayer against surface oxidation under working conditions.

## 4. Conclusions

In summary, the potential of several 2D Si*_x_*C*_y_* monolayers as metal-free NO_3_ER catalysts for NH_3_ synthesis was evaluated by performing comprehensive density functional theory computations. Our results showed that the unique structure of the Si_3_C monolayer can sufficiently activate the NO_3_^−^ reactant by forming a “3−O” adsorption configuration. Moreover, the adsorbed ^*^NO_3_ intermediate can be easily hydrogenated into a NH_3_ product due to the excellent synergistic effect of the three Si active sites with a low limiting potential of −0.43 V. Meanwhile, the high free energy barriers hinder the formation of NO_2_, NO, and N_2_ byproducts, and the considerable positive charges on the Si active sites result in weaker H adsorption than NO_3_^−^, ensuring more favorable NO_3_ER than the competitive HER. In addition, the Si_3_C monolayer exhibits a low formation energy in Si-rich conditions and good stability, providing great potential for its experimental synthesis and practical application. Remarkably, acidic conditions are beneficial to promote the NO_3_^−^-to-NH_3_ conversion. The low limiting potential, high selectivity, great promise for synthesis, and high stability render the Si_3_C monolayer as a very compelling electrocatalyst for NO_3_^−^ electrochemical reduction, which may offer new opportunities for efficient nitrate removal and NH_3_ synthesis by using metal−free catalysts.

## Data Availability

The data presented in this study are available on request from the corresponding author.

## Supplementary Materials

The following supporting information can be downloaded at: https://www.mdpi.com/article/10.3390/nano13212890/s1, Figure S1. The optimized structures, charge density distribution, and the band structures of (a) SiC, (b) SiC_2_, (c) SiC_3_, (d) SiC_5_, and (e) SiC_7_ monolayers. The isovalue was set to 0.003 e Å^−3^, and yellow and red bubbles represent positive and negative charges, respectively. The Fermi level was set to zero in red dotted lines; Figure S2. The most stable NO_3_^*^ adsorption configurations were viewed from the top and side of these catalysts of (a) SiC, (b) SiC_2_, (c) SiC_3_, (d) SiC_5_, and (e) SiC_7_ monolayers; Figure S3. The integrated-crystal orbital Hamilton population (ICOHP) about N-O which from NO_3_^*^ is adsorbed species on (a) SiC, (b) SiC_2_, (c) SiC_3_, (d) SiC_5_, (e) SiC_7_ and (f) Si_3_C monolayers; Figure S4. The involved configurations of intermediates for NO3ER on Si_3_C monolayer; Figure S5. The kinetic process for the dissociation of NO_3_^*^ with the help of H^+^ to NO_2_^*^ + OH^*^ on Si_3_C monolayer; Figure S6. The obtained free energy diagrams of NO_3_ER on (a) SiC_3_ and (b) SiC_7_ catalysts; Figure S7. The free energy diagram of NO_3_ER on Si_3_C obtained by rPBE method; Figure S8. The obtained free energy diagram of N_2_ formation on Si_3_C monolayer.; Figure S9. The involved free energy profile for NO_3_-to-NH_3_ electrocatalytic reduction on Si_3_C monolayer at pH = 2.90; Figure S10. (a) Variations of temperature and energy as a function of the time for AIMD simulations and (b) Pourbaix profile of Si_3_C monolayer; Table S1. The correction of zero-point energy (ZPE, eV) and entropy (TS, eV) of molecules involved in NO_3_ER. T is set to 298.15 K; Table S2. The optimized lattice parameters of unit cells (l, Å), lengths of Si-C bonds (d_Si-C_, Å), C-C bonds (d_C-C_, Å), and Si-Si bonds (d_Si-Si_, Å), formation energies under C-rich (E_f-C_, eV) and Si-rich conditions (E_Si-C_, eV), the charge transfer (Q, e) from Si atoms to C atoms, and the band gaps (Egap, eV) of 2D SixCy monolayers; Table S3. The data on the optimized structure of Si_3_C nanomaterial were compared with those in the literature; Table S4. The all computed free energy changes (∆G, eV) of each elementary step of Si3C monolayer. The ∆G values of the selected steps are remarked in red; Table S5. The quadratic relation between the energy (E) of the reaction intermediates and de-pendence of applied electrochemical potential U.
