# Supplementary material for: Si3C Monolayer as an Efficient Metal-Free Catalyst for Nitrate Electrochemical Reduction: A Computational Study
Source: Nanomaterials (Basel). 2023 Oct 31;13(21):2890. doi: 10.3390/nano13212890 (PMC10649319; doi:10.3390/nano13212890)
Supplement: Supplementary file 1 [file nanomaterials-13-02890-s001.zip › nanomaterials-2672118-supplementary.pdf]

nanomaterials

MDPI

Supplementary Materials

Si_3C Monolayer as an Efficient Metal-Free Catalyst for Nitrate Electrochemical Reduction: A Computational Study

Wanying Guo [1], Tiantian Zhao [1], Fengyu Li [2,*], Qinghai Cai [1] and Jingxiang Zhao [1,*]

[1] College of Chemistry and Chemical Engineering, and Key Laboratory of Photonic and Electronic Bandgap Materials, Ministry of Education, Harbin Normal University, Harbin 150025, China; guowanying@stu.hrbnu.edu.cn (W.G.); zhaotiantian@stu.hrbnu.edu.cn (T.Z.); caiqinghai@hrbnu.edu.cn (Q.C.)
[2] School of Physical Science and Technology, Inner Mongolia University, Hohhot 010021, China
* Correspondence: fengyuli@imu.edu.cn (F.L.); zhaojingxiang@hrbnu.edu.cn (J.Z.)

COMPUTATIONAL DETAILS

Constant-Potential Computations

In the constant-potential calculations, to clarify the reaction mechanism at differernt electrode potentials, we changed the excess charge per unit cell (Δn) from − 2.0 e^- to + 2.0 e^-, with a step of 0.5 e^-. The potential related energy of the system is defined as follows:

$$E = E_{DFT} - \Delta n(V_{sol} + \varphi_q/e)$$

where E_{DFT} is the DFT-calculated energy, V_{sol} is the electrostatic potential of the bulk electrolyte, and $-\varphi_q$ is the work function of the charged system.

The electric potential of the slab referenced to the SHE is calculated as:

$$U_q(U/SHE) = -4.6 - \varphi_q(f)\ (eV)$$

where $-\varphi_q(f)$ is the work function of the charged slab in aqueous solution and 4.6 V is the work function of the H_2 /H^+ couple at standard conditions. In fact, the work function measured by SHE is dispersed from 4.4 to 4.8 eV, and our calculation took the average of 4.6 eV.

The E-Uq quadratic form could be written as:

$$E(U_q) = -\frac{1}{2}C\left(U_q - U_0\right)^2 + E_0$$

where U_0, C, and E_0 refer to the potential of zero charge (PZC), capacitance of the corresponding system, and the energy of the system at the PZC, respectively.

Fixed potential can be calculated by changing the potential reference value of pH value through the following method:

$$U_{RHE} = U_{SHE} + 0.0592 \times pH$$

In addition, the relationship between the adsorption energy (E_{ads}) of the reaction intermediate and pH can be derived as follows. In detail, the relationship between the total electronic energy (E_{int*}, eV) of a given reaction intermediate and the U_{SHE} can be described by: E_{int*} (eV) = a×U_{SHE}^2+ b×U_{SHE}+c, where a, b, and c represent the computed coefficients in Table S5, U_{SHE} can be obtained by: $U_{SHE} = U_{RHE} - 0.0592 \times pH$, which is pH-dependent. Moreover, the adsorption energy (E_{ads}, eV) of a given reaction intermediate can be determined by: E_{ads} (eV) = E_{int*} E_* E_{int}, where E_* and E_{int} are the total electronic energies of catalyst and a free intermediate, respectively.

Table S1. The correction of zero-point energy (ZPE, eV) and entropy (TS, eV) of molecules involved in NO_3ER. T is set to 298.15 K.

	**ZPE**	**TS**
NO_3^*	0.33	0.00
$NO_2^* + OH^*$	0.61	0.00
NO_2^*	0.23	0.00
$NO^* + OH^*$	0.53	0.00
NO^*	0.17	0.00
$N^* + OH^*$	0.48	0.00
$NH^* + OH^*$	0.72	0.00
$NH_2^* + OH^*$	1.05	0.00
OH^*	0.36	0.00
NO_2 (g)	0.23	0.74
NO_3H (g)	0.55	0.77
NO (g)	0.12	0.64
N_2O (g)	0.30	0.70
N_2 (g)	0.15	0.59
NH_3 (g)	0.90	0.59
H_2 (g)	0.27	0.41
H_2O (g)	0.56	0.68

Table S2. The optimized lattice parameters of unit cells (l, Å), lengths of Si-C bonds (d_{Si-C}, Å), C-C bonds (d_{C-C}, Å), and Si-Si bonds (d_{Si-Si}, Å), formation energies under C-rich (E_{f-C}, eV) and Si-rich conditions (E_{Si-C}, eV), the charge transfer (Q, e) from Si atoms to C atoms, and the band gaps (E_{gap}, eV) of 2D Si_xC_y monolayers.

Si_xC_y	$l_a = l_b$	d_{Si-C}	d_{C-C}	d_{Si-Si}	E_{f-C}	E_{f-Si}	Q	E_{gap}
SiC	3.09	1.78	/	/	0.67	0.67	2.44	2.56
SiC_2	5.00	1.80	1.44	/	0.66	0.86	2.42	0.61
SiC_3	5.62	1.81	1.44	/	0.63	0.93	2.22	/
SiC_5	4.64	1.76	1.45	/	0.52	0.93	2.51	/
SiC_7	5.29	1.69	1.44	/	0.58	1.04	2.51	0.76
Si_3C	7.02	1.81	/	2.45	1.20	0.90	0.74	/

Table S3. The data on the optimized structure of Si_3C nanomaterial were compared with those in the literature.

Si_3C	$l_a = l_b$	d_{Si-C}	d_{Si-Si}	E_{gap}
Our result	7.02	1.81	2.45	0
Ref. 41	7.02	1.81	2.25	/
Ref. 45	7.04	/	2.26	0

Table S4. The all computed free energy changes (ΔG, eV) of each elementary step of Si_3C monolayer. The ΔG values of the selected steps are remarked in red.

Elementary step	ΔG
$HNO_3 + {}^* \rightarrow NO_3^* + H^+$	−0.26
$NO_3^* + H^+ + e^- \rightarrow NO_2^* + OH^*$	−1.12
$NO_2^* + OH^* + H^+ + e^- \rightarrow NO_2^* + H_2O$	0.32
$NO_2^* + H^+ + e^- \rightarrow NO^* + OH^*$	−1.90
$NO_2^* + H^+ + e^- \rightarrow NHO_2^*$	−0.60
$NO^* + OH^* + H^+ + e^- \rightarrow NO^*(side) + H_2O$	0.37
$NHO_2^* + H^+ + e^- \rightarrow NHO^* + OH^*$	−2.05
$NO^* + OH^* + H^+ + e^- \rightarrow NO^*(end) + H_2O$	1.07
$NO^*(side) + H^+ + e^- \rightarrow N^* + OH^*$	−2.54
$NO^*(side) + H^+ + e^- \rightarrow NHO^*$	−0.36
$NHO^* + OH^* + H^+ + e^- \rightarrow NHO^* + H_2O$	0.36
$NHO^* + OH^* + H^+ + e^- \rightarrow NH_2O^* + OH^*$	−0.17
$NO^*(end) + H^+ + e^- \rightarrow NOH^*$	0.29
$NO^*(end) + H^+ + e^- \rightarrow NHO^*$	0.34
$N^* + OH^* + H^+ + e^- \rightarrow NH^* + OH^*$	−0.62
$N^* + OH^* + H^+ + e^- \rightarrow N^* + H_2O$	0.67
$NHO^* + H^+ + e^- \rightarrow NH_2O^*$	−0.01
$NHO^* + H^+ + e^- \rightarrow NH^* + OH^*$	−2.56
$NH_2O^* + OH^* + H^+ + e^- \rightarrow NH_2O^*$	0.56
$NOH^* + H^+ + e^- \rightarrow N^* + H_2O$	−2.89
$NHO^* + H^+ + e^- \rightarrow NHOH^*$	−1.03
$NH^* + OH^* + H^+ + e^- \rightarrow NH_2^* + OH^*$	−0.07
$NH_2O^* + H^+ + e^- \rightarrow O^* + H_2O$	−2.61
$N^* + H^+ + e^- \rightarrow NH^*$	−0.78
$NHOH^* + H^+ + e^- \rightarrow NH^* + H_2O$	−2.64
$NH_2^* + OH^* + H^+ + e^- \rightarrow OH^* + NH_3$	0.16
$O^* + H^+ + e^- \rightarrow OH^*$	0.27
$NH^* + H^+ + e^- \rightarrow NH_2^*$	0.26
$OH^* + H^+ + e^- \rightarrow H_2O + {}^*$	0.43
$NH_2^* + H^+ + e^- \rightarrow NH_3^*$	0.02
$NH_3^* \rightarrow NH_3 + {}^*$	−0.24

Table S5. The quadratic relation between the energy (E) of the reaction intermediates and dependence of applied electrochemical potential U.

Reaction intermediates	Energy (E)	R^2
*	$E = -1.26U^2 - 0.91U - 417.14$	0.98
NO_3^*	$E = -2.71U^2 - 1.21U - 443.74$	0.96
$NO_2^* + OH^*$	$E = -2.81U^2 - 1.54U - 448.52$	0.99
NO_2^*	$E = -2.44U^2 - 1.03U - 436.95$	0.99
$NO^* + OH^*$	$E = -2.09U^2 - 0.86U - 442.58$	0.98
NO^*	$E = -1.45U^2 - 0.48U - 430.94$	0.99
$N^* + OH^*$	$E = -2.09U^2 - 1.32U - 437.61$	0.97
$NH^* + OH^*$	$E = -1.55U^2 - 0.81U - 441.68$	0.99
$NH_2^* + OH^*$	$E = -1.49U^2 - 1.29U - 445.75$	0.94
OH^*	$E = -1.71U^2 - 1.02U - 428.91$	0.99

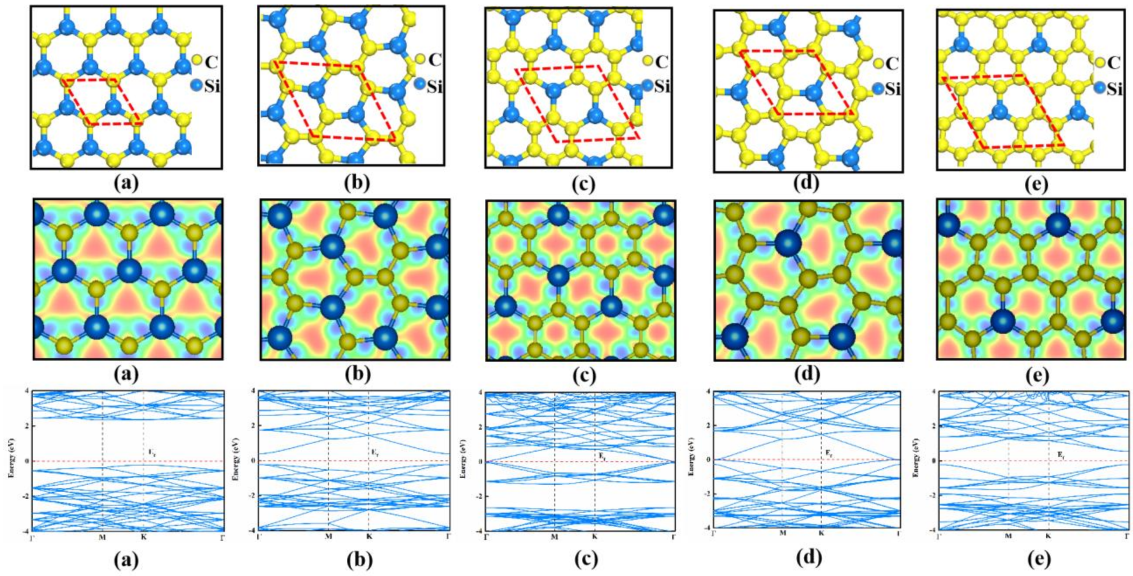

Figure S1. The optimized structures, charge density distribution, and the band structures of (**a**) SiC, (**b**) SiC_2, (**c**) SiC_3, (**d**) SiC_5, and (**e**) SiC_7 monolayers. The isovalue was set to 0.003 e Å^{-3}, and yellow and red bubbles represent positive and negative charges, respectively. The Fermi level was set to zero in red dotted lines.

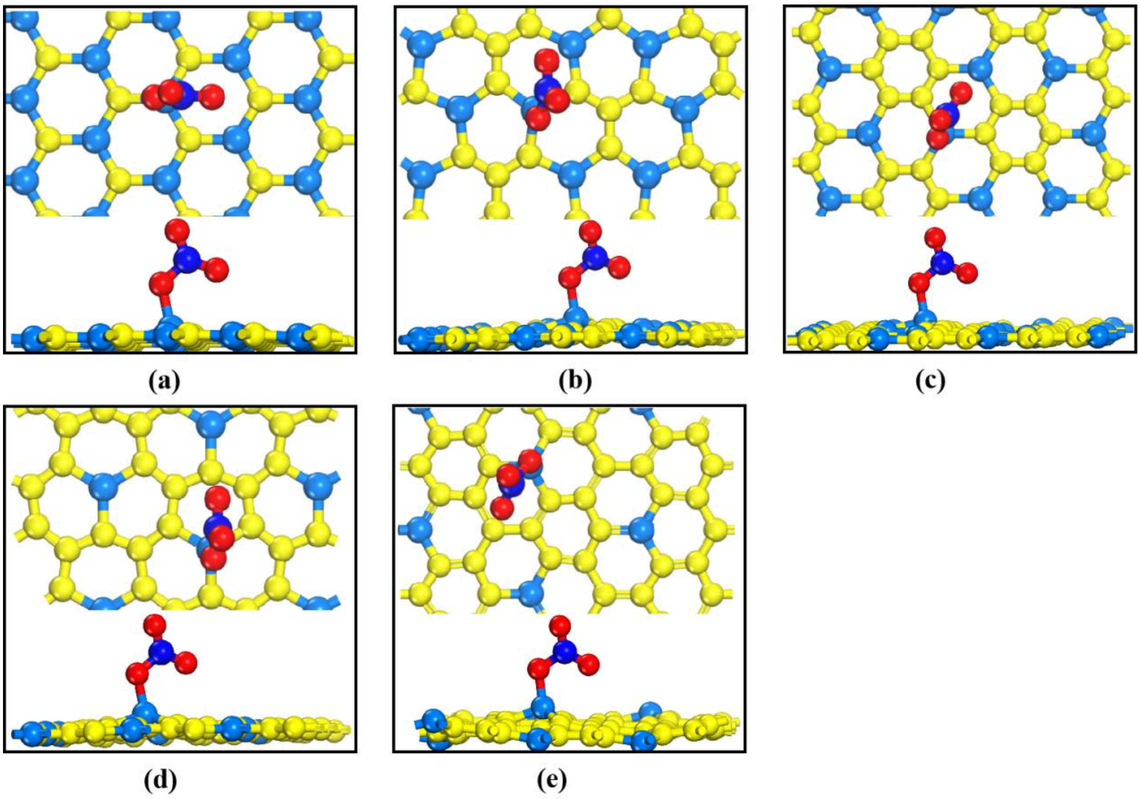

Figure S2. The most stable NO_3^* adsorption configurations were viewed from the top and side of these catalysts of (**a**) SiC, (**b**) SiC_2, (**c**) SiC_3, (**d**) SiC_5, and (**e**) SiC_7 monolayers.

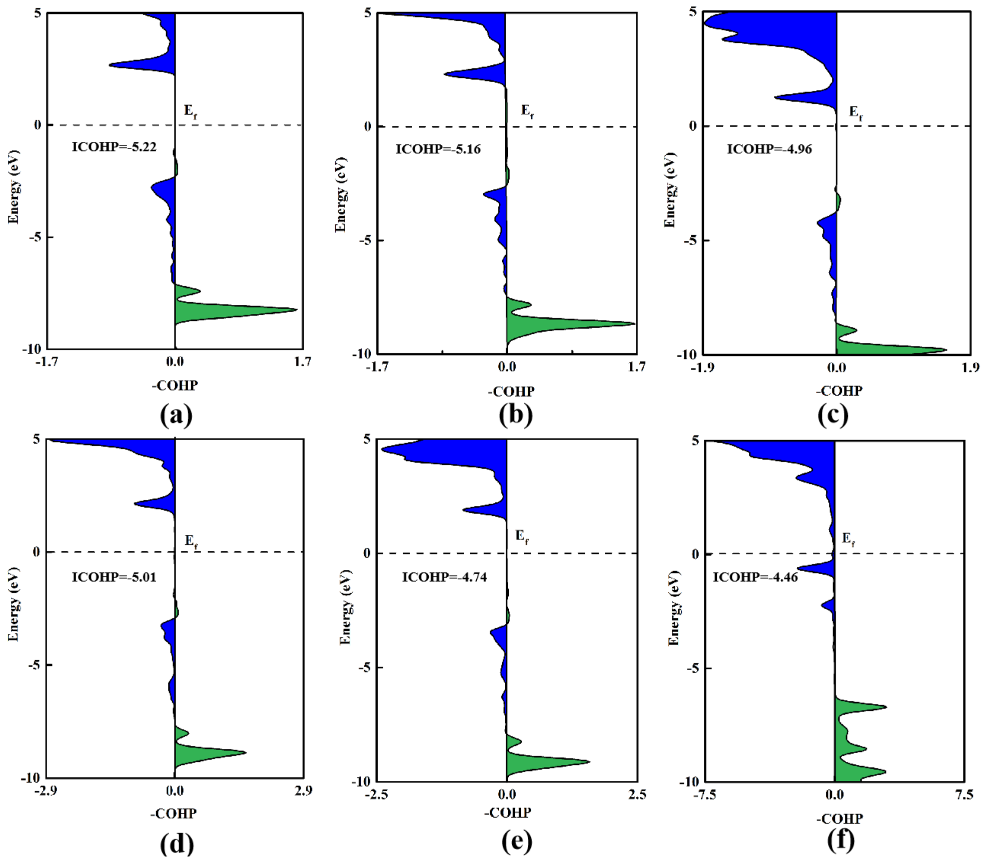

Figure S3. The integrated-crystal orbital Hamilton population (ICOHP) about N-O which from NO_3^* is adsorbed species on (**a**) SiC, (**b**) SiC_2, (**c**) SiC_3, (**d**) SiC_5, (**e**) SiC_7 and (**f**) Si_3C monolayers.

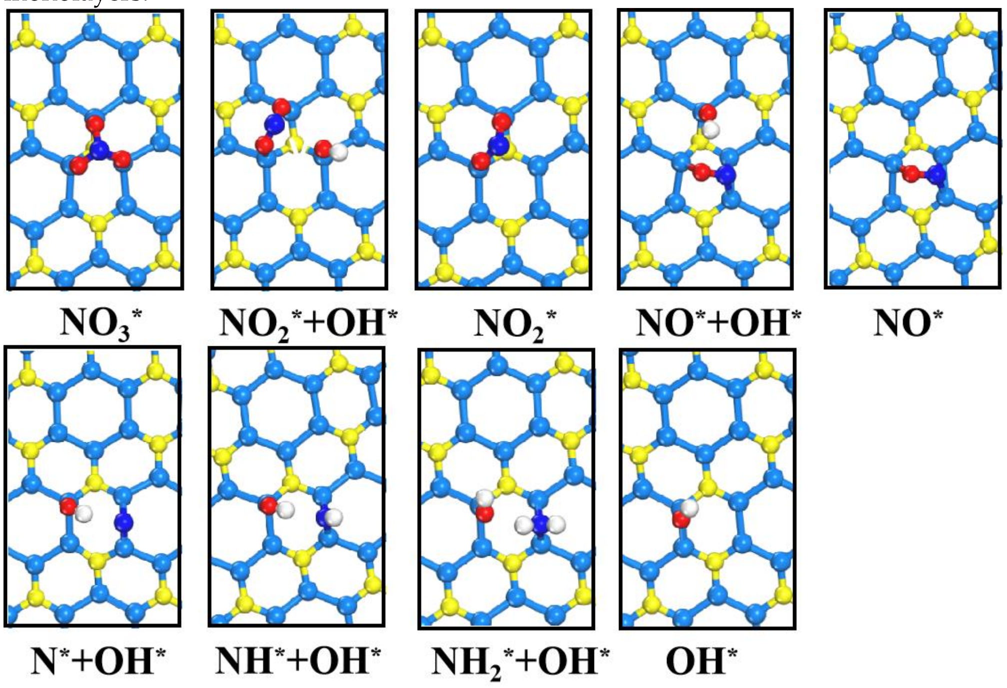

Figure S4. The involved configurations of intermediates for NO_3ER on Si_3C monolayer.

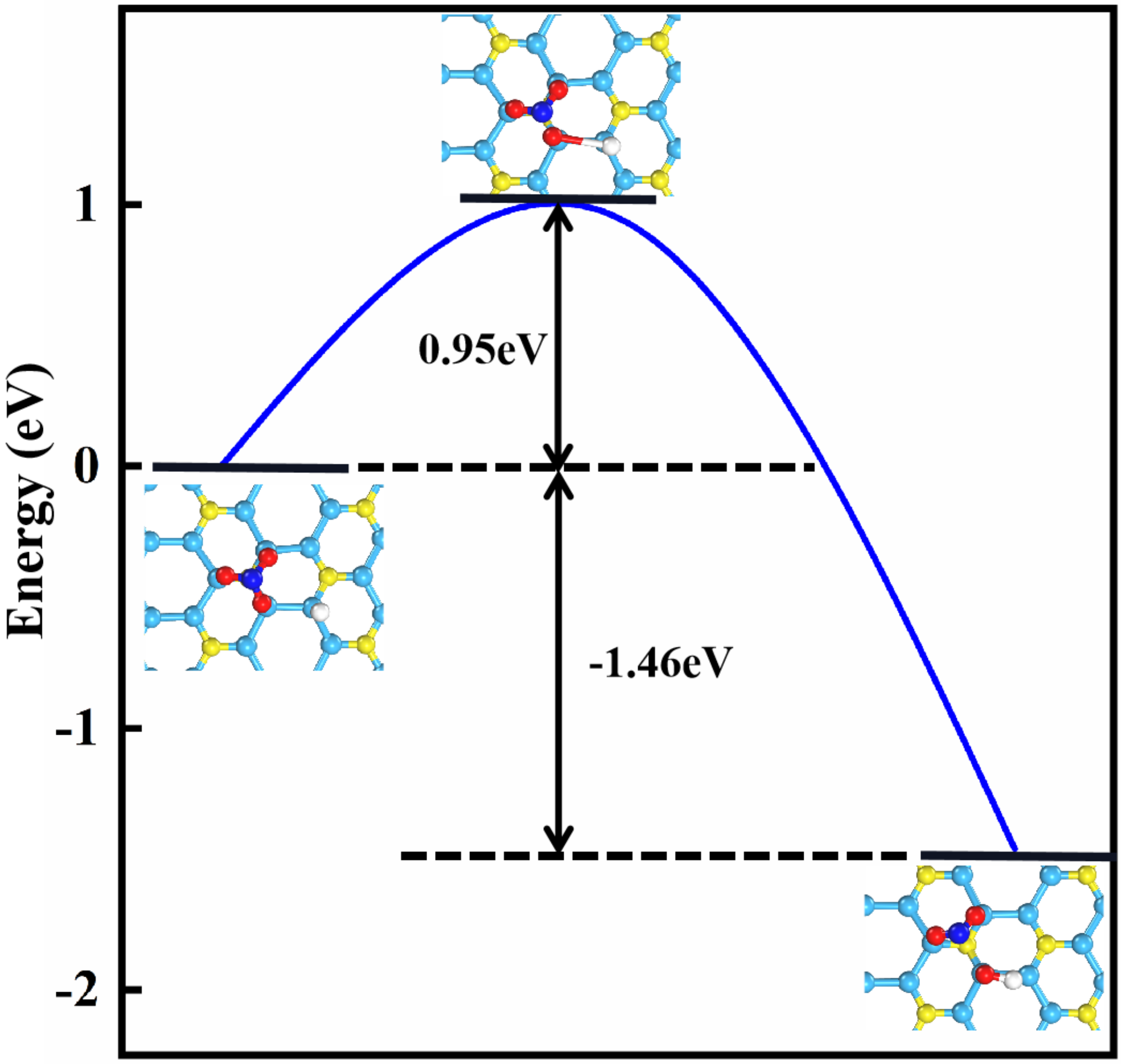

Figure S5. The kinetic process for the dissociation of NO_3^* with the help of H^+ to NO_2^* + OH^* on Si_3C monolayer.

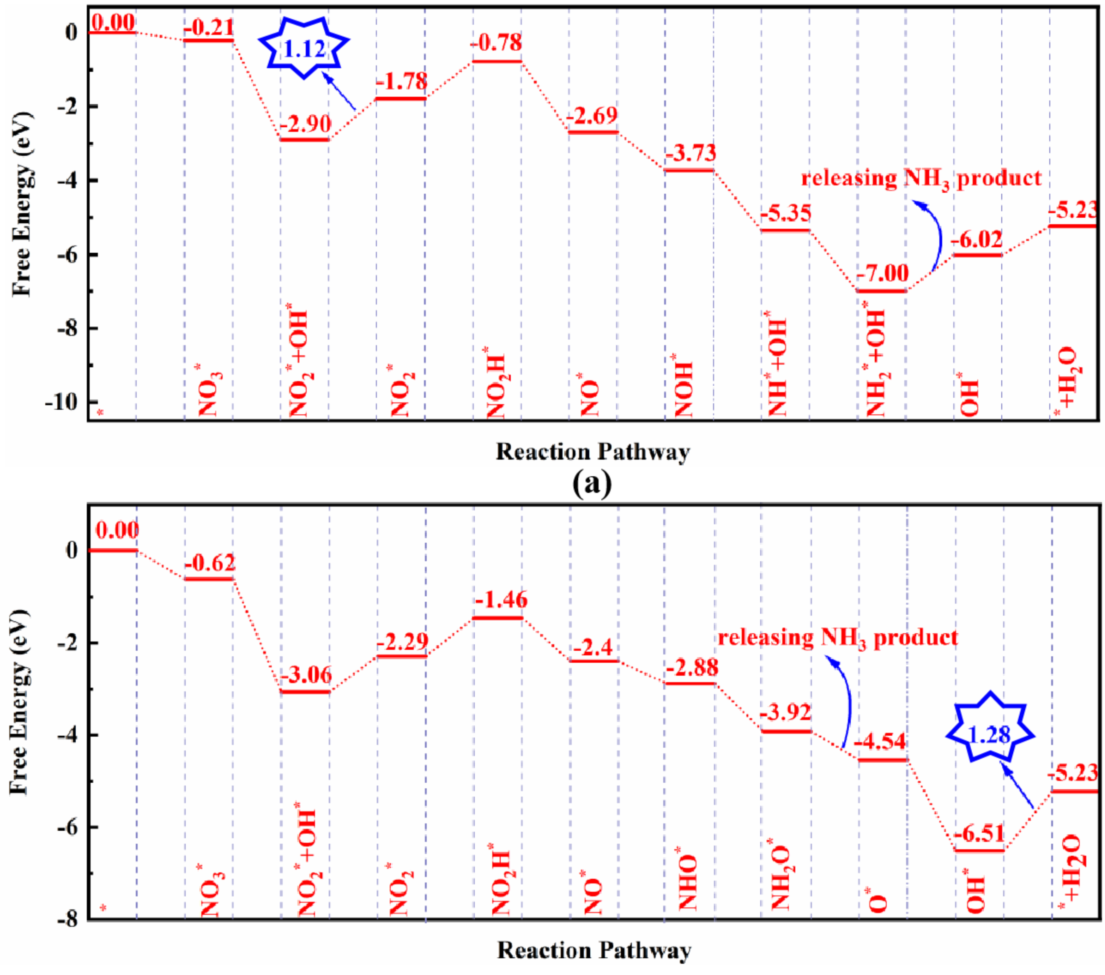

(b)

Figure S6. The obtained free energy diagrams of NO3ER on (**a**) SiC3 and (**b**) SiC7 catalysts.

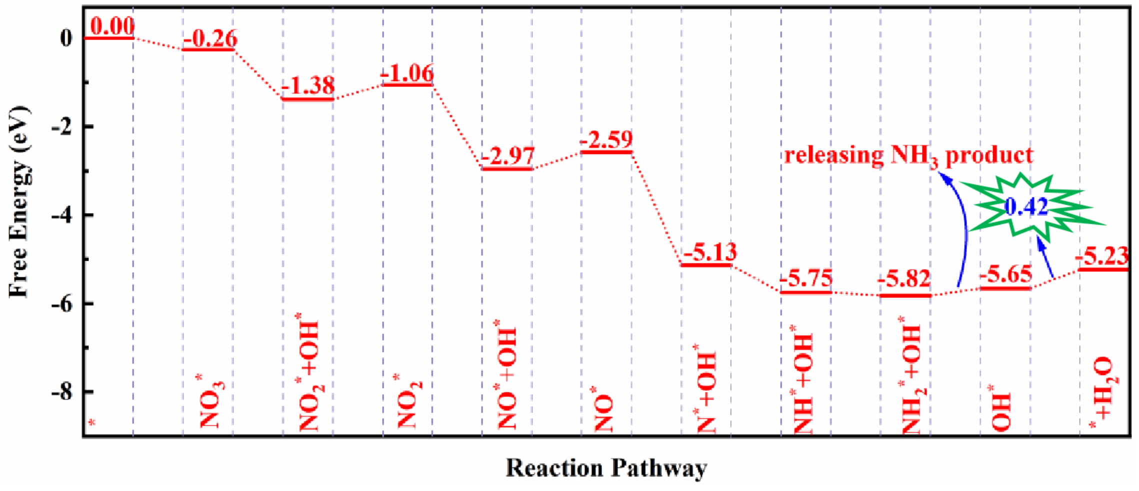

Figure S7. The free energy diagram of NO3ER on Si3C obtained by rPBE method.

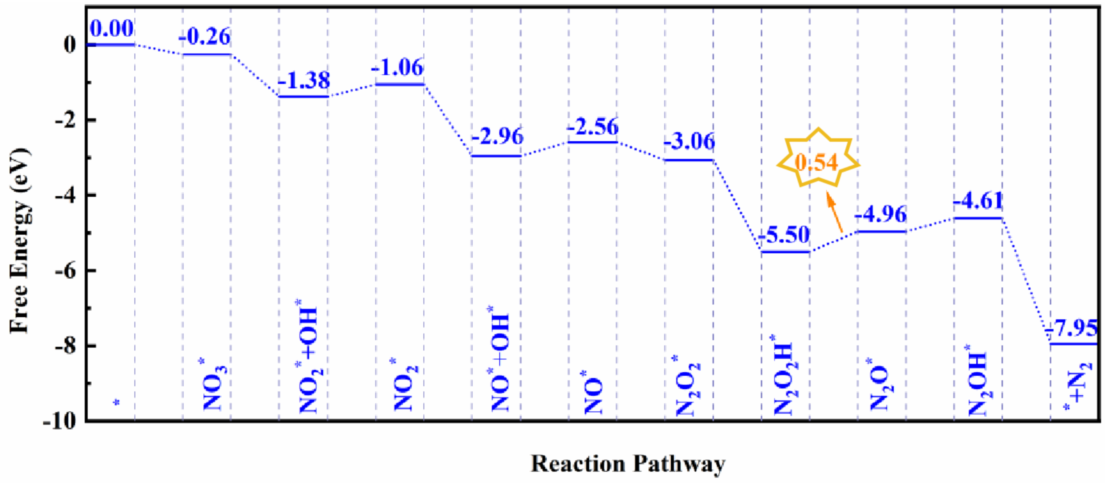

Figure S8. The obtained free energy diagram of N_2 formation on Si_3C monolayer.

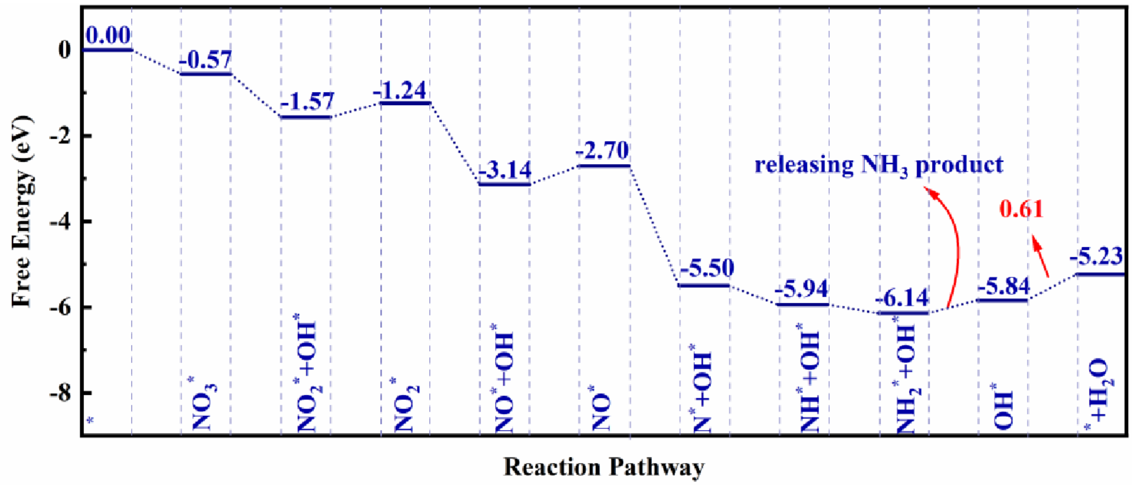

Figure S9. The involved free energy profile for NO_3-to-NH_3 electrocatalytic reduction on Si_3C monolayer at pH = 2.90.

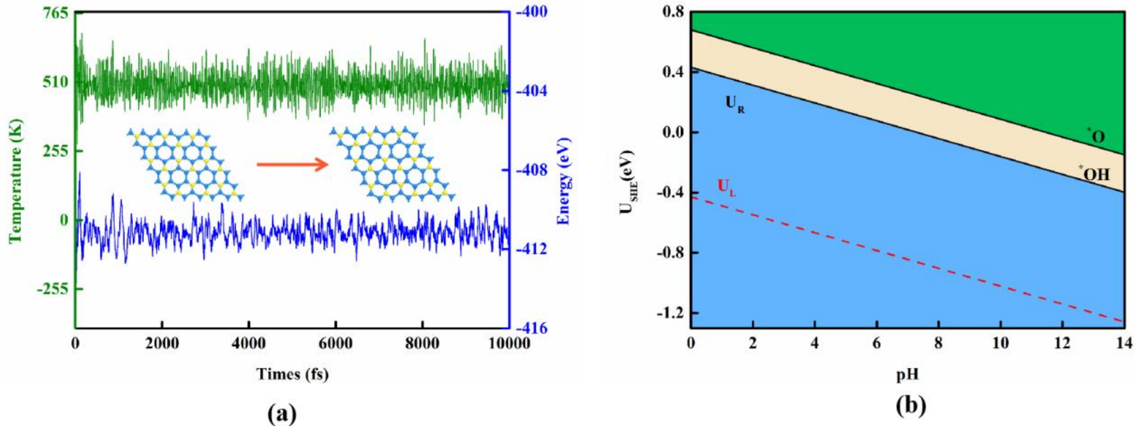

Figure S10. (**a**) Variations of temperature and energy as a function of the time for AIMD simulations and (**b**) Pourbaix profile of Si_3C monolayer.
